# Editorial: Bio-Technological Processes and Enzymes for the Conversion and Valorization of Plastic Wastes

**DOI:** 10.3389/fbioe.2022.873068

**Published:** 2022-03-24

**Authors:** Evangelos Topakas, Jasmina Nikodinovic-Runic, Qingsheng Qi

**Affiliations:** ^1^ Industrial Biotechnology and Biocatalysis Group, Biotechnology Laboratory, School of Chemical Engineering, National Technical University of Athens, Athens, Greece; ^2^ Eco-Biotechnology and Drug Development Group, Laboratory for Microbial Molecular Genetics and Ecology, Institute of Molecular Genetics and Genetic Engineering, University of Belgrade, Belgrade, Serbia; ^3^ State Key Laboratory of Microbial Technology, Shandong University, Qingdao, China

**Keywords:** synthetic polymer converting enzymes, pretreatment processes, upcycling plastic wastes, discovery of novel enzymes, biocatalysis, consolidated processes

Plastics are ubiquitous and indispensable materials in the world economy and our daily lives, providing both high performance and energy saving benefits along with alarming pollution and waste stockpiles. The predominant consumer petroleum-based synthetic polymers, such as low-density polyethylene, high-density polyethylene, polyvinyl chloride, polystyrene, and polypropylene, poly(ethylene terephthalate) (PET), and polyurethanes, take hundreds of years to degrade in the environment, leading to a long-lasting blight on our oceans, countryside, and now even in our food system. As the production of plastic is expected to double over the next 20 years, plans such as legislation requiring all plastic packaging within the EU market to be either reusable or recyclable in a cost-effective manner by 2030 are increasingly important.

It is now widely recognized that changing from consumption and transitioning to sustainable growth models is essential to safeguarding the planet and people. The development of new regenerative technologies is essential to eliminating the indelible imprint of pervasive plastic, to deliver plastics circularity, and secure the future prosperity of the planet and its inhabitants. Pioneering technologies and innovative scientific developments are essential in the mission to transition from the linear to the circular plastic economy. Nature’s biodegradation and bioregeneration processes combine environmental weathering and microbial and enzymatic activities for depolymerization of post-use macromolecules into constituent building blocks. Biochemical synthesis and repolymerization have the potential to revalorize these building-block molecules as functional bioconstructs to complete the loop and enable continuous life cycle operation for the next generation of plastic materials and products. Our challenge is now to effectively conjugate the necessary cross-disciplinary activities and progress the required science and engineering to complete the life-cycle for plastics.

For the development of eco-sustainable plastic degradation and recycling processes, both bacteria and fungi are becoming a major microbial resource. In the study published by Quartinello et al., the rumen content of *Bos taurus* was investigated regarding its potential as a synthetic polyester degrading enzymatic system. The rumen’s enzymatic cocktail was screened using a series of model chromophoric substrates, as well as two synthetic terephthalate polyesters. Additionally, the well-known biobased polyester poly(ethylene furanoate) (PEF) was also hydrolyzed by rumen fluid, as it was justified by HPLC and SEM analysis. Metagenomic analysis revealed that the rumen microbiome capable of hydrolyzing the synthetic polyesters was dominated by bacteria (98%) presenting a small proportion of Eukayota (1%) and Archaea (1%).

In the field of synthetic polyester degradation, the broadly used petroleum-based and non-biodegradable PET was efficiently hydrolyzed by an engineered cutinase isolated by leaf-branch compost metagenome (LCC). To improve the enzyme polyester degradation performance, Xue et al. fused a chitin-binding domain (ChBD) from *Chitinolyticbacter meiyuanensis* SYBC-H1 to the C-terminus of a previously reported LCC variant. The fused enzyme demonstrated higher adsorption to the polyester substrate, overall improving the degradation performance by 20% compared to the precursor enzyme without the binding module. Additionally, different binding domains were fused to the LCC variant and tested against PET substrates with various crystallinity degrees, indicating that fusing a polymer-binding module to a polyesterase is a promising method for stimulating the enzymatic hydrolysis of PET.

Aiming to enhance PET degrading enzymes, a different approach was followed by Haernvall et al. through the exchange of the canonical amino acid methionine in the active site of *Thermoanaerobacter thermohydrosulfuricus* lipase (TLL) by the more hydrophobic non-canonical norleucine. Structural modelling of TTL revealed target methionine residues to be replaced by norleucine that resulted in a significant enhancement of PET hydrolysis. A similar trend was also observed for the hydrolysis of an ionic phthalic polyester containing a short alkyl diol (C5). Two-fold increase of the modified TTL towards ionic phthalic polyesters containing different ether diols compared to the parent enzyme was achieved, demonstrating the potential of non-canonical amino acids for enzyme engineering.

Last but not least, a review article by Nikolaivits et al. summarized the mechano-biocatalytic approaches for waste plastic (re)valorization. A number of mechanical and green chemical (pre)treatment methodologies were reviewed, including mechanical milling, reactive extrusion, ultrasonic-, UV-, as well as supercritical CO_2_. Such processes render the plastic materials more amenable to microbial and enzymatic attack for the effective depolymerization and subsequent (re)valorization. A comprehensive overview of the reported microbial and enzymatic degradation of petroleum-based synthetic polymer plastics is detailed, while the harvesting of depolymerization products to produce new materials and higher-value products was highlighted.

All Topic Editors would like to express their gratitude to all the authors for their valuable contributions, as well as to the editorial team of Frontiers for their kind support.

